# Use of archival data on Indian residential schools

**DOI:** 10.3402/ijch.v75.32591

**Published:** 2016-06-27

**Authors:** Tracey Galloway, Rhonda Johnson

**Affiliations:** 1 International Journal of Circumpolar Health, University of Toronto Mississauga, Mississauga, ON, Canada. Email: tracey.galloway@utoronto.ca; 2 International Journal of Circumpolar Health, University of Alaska Anchorage, Anchorage, AK, USA. Email: rmjohnson2@uaa.alaska.edu

Library and Archives Canada (LAC) contains a number of resources relevant to Indigenous heritage, among them the School Files Series which contains government records created from 1879 to 1953 about residential and day schools operating in Canada and available on microfilm. In recent years, many of these microfilm images have been digitized and made available on the LAC and other websites.

The school records contain information on admissions, discharges, day-to-day operation and financial reports. They also contain material on family background, health examinations and death registers. The material is organized using a series of filing systems and is accompanied by helpful guides and search tools. Much of the data are “open-coded” meaning the material is available for public use. Researchers may freely consult these records without restriction.

The Tri-Council Policy Statement on Research Involving Humans states that research involving publicly available data is exempt from the requirement for ethical review. However, the statement also recommends that researchers interpreting the ethical framework in Indigenous contexts uphold the principles of respect for persons, concern for welfare and the advancement of justice.

To that end, we have asked the authors of this paper to provide a brief commentary on the ethical issues arising from their work. We have also asked Ry Moran, Director of the National Centre for Truth and Reconciliation, to comment on the implications of this type of research for the development and implementation of processes surrounding data access and use.

*Tracey Galloway* Deputy Editor International Journal of Circumpolar Health University of Toronto Mississauga Mississauga, ON, Canada Email: tracey.galloway@utoronto.ca *Rhonda Johnson* Editor-in-Chief International Journal of Circumpolar Health University of Alaska Anchorage Anchorage, AK, USA Email: rmjohnson2@uaa.alaska.edu

